# Molecular Dynamics Investigation of Clustering in Aqueous Glycine Solutions

**DOI:** 10.1021/acs.jpcb.2c01975

**Published:** 2022-06-21

**Authors:** Martin B. Sweatman, Nasser D. Afify, Carlos A. Ferreiro-Rangel, Miguel Jorge, Jan Sefcik

**Affiliations:** † School of Engineering, The University of Edinburgh, The King’s Buildings, Sanderson Building, Mayfield Road, Edinburgh EH9 3JL, U.K.; ‡ Department of Chemical and Process Engineering, Faculty of Engineering, University of Strathclyde, James Weir Building, Montrose Street, Glasgow G1 1XJ, U.K.

## Abstract

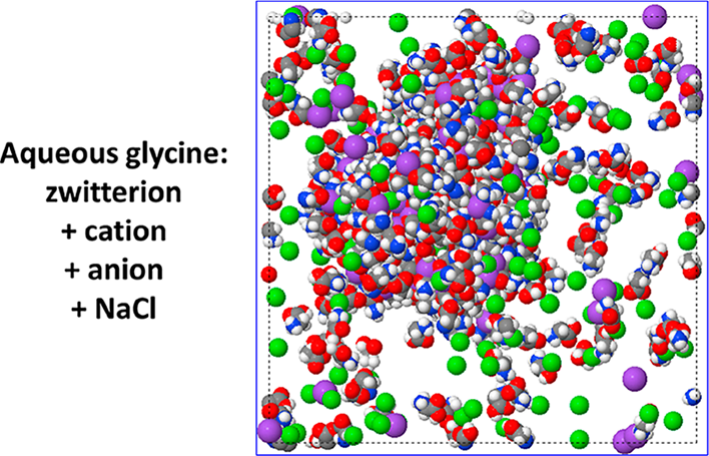

Recent experiments with undersaturated aqueous glycine solutions have repeatedly exhibited the presence of giant liquid-like clusters or nanodroplets around 100 nm in diameter. These nanodroplets re-appear even after careful efforts for their removal and purification of the glycine solution. The composition of these clusters is not clear, although it has been suggested that they are mainly composed of glycine, a small and very soluble amino acid. To gain insights into this phenomenon, we study the aggregation of glycine in aqueous solutions at concentrations below the experimental solubility limit using large-scale molecular dynamics simulations under ambient conditions. Three protonation states of glycine (zwitterion = GLZ, anion = GLA, and cation = GLC) are simulated using molecular force fields based on the 1.14*CM1A partial charge scheme, which incorporates the OPLS all-atom force field and TIP3P water. When initiated from dispersed states, we find that giant clusters do not form in our simulations unless salt impurities are present. Moreover, if simulations are initiated from giant cluster states, we find that they tend to dissolve in the absence of salt impurities. Therefore, the simulation results provide little support for the possibility that the giant clusters seen in experiments are composed purely of glycine (and water). Considering that strenuous efforts are made in experiments to remove impurities such as salt, we propose that the giant clusters observed might instead result from the aggregation of reaction products of aqueous glycine, such as diketopiperazine or other oligoglycines which may be difficult to separate from glycine using conventional methods, or their co-aggregation with glycine.

## Introduction

Glycine is the simplest amino acid and an important building block of proteins. It is essential for life and has even been detected in deep space.^1^ Given its fundamental role in biochemical processes, it is essential that we understand its basic physicochemical behavior, which includes its behavior in aqueous solutions.

With this in mind, it is worth considering recent experimental observations of large colloidal-scale aggregates in undersaturated aqueous glycine solutions that defy initial explanation.^2,3^ In this earlier work, all attempts to permanently remove these large aggregates by purifying the glycine solution apparently failed, which suggests that these giant clusters, or nanodroplets, are an equilibrium phase of aqueous glycine itself rather than an impurity. If true, this behavior is not understood. Attempts to purify these aqueous glycine solutions included nanofiltration to remove colloidal-scale objects and repeated stages of re-crystallization and washing with deionized water to remove remaining impurities. Nevertheless, even after these efforts, nanodroplets re-appear in these solutions after a lengthy nucleation period, sometimes lasting days. However, nucleation of these nanodroplets can occur after less than 1 h of vigorous stirring.

In turn, much of this prior work on clustering in aqueous glycine solutions was motivated by the study of non-classical nucleation,^4−6^ whereby the crystal phase nucleates from solution via an intermediate non-crystalline microphase, typically a liquid-like nanodroplet. Glycine is viewed as an ideal model for these studies, given that it appears to exhibit these intermediate liquid-like structures close to saturation and is a relatively simple, small molecule. Moreover, work focused on the nucleation of crystalline glycine from aqueous glycine solutions shows that several factors can influence the resulting polymorph formed, including additives and evaporation rates.^7^ Very interestingly, the formation of nanodroplets in supersaturated glycine solutions can also be triggered by laser irradiation.^8−10^

In fact, giant equilibrium clusters in solution are observed for many solute–solvent systems.^11^ Typically, these involve large, charged solutes that form even larger charge-stabilized clusters with a specific average size. Examples include many kinds of polyelectrolytes, including proteins^12,13^ and peptides.^14^

In many cases, the formation of giant equilibrium clusters can be understood in terms of the SALR (short-range attraction and long-range repulsion) model fluid.^15,16^ Although details of the global phase behavior of the SALR model are still actively researched, it is known to form giant clusters at a low concentration under suitable conditions. These clusters are size-limited because growth of a bulk liquid phase is arrested by the accumulation of repulsive (typically screened coulomb) interactions as the cluster size increases.

Since the length scale is arbitrary in the SALR model, there is no reason in principle why small molecules with effective SALR interactions in solution should not also form giant clusters like their much larger counterparts. However, in such cases, the clusters themselves are likely to be smaller, perhaps too small in many cases even for optical microscopy to image, and therefore, the presence of giant equilibrium clusters in many small-molecule solutions, similar to aqueous glycine, might have gone unnoticed.

This is the context of this work. Glycine is considered a very small and highly soluble molecule which mainly forms zwitterionic species in solution at near-neutral pH. It is, therefore, usually thought unlikely to form giant equilibrium clusters in undersaturated solutions. Nevertheless, large, persistent, and apparently equilibrium clusters or nanodroplets are observed in experiments with aqueous glycine. They appear to be sensitive to experimental conditions and influence glycine crystallization nucleation rates and the resulting glycine polymorph. The key question, which this work seeks to address, therefore, is whether these clusters are composed mainly of glycine or an impurity. Moreover, is the SALR mechanism responsible for their formation? Insights into these questions could lead to progress in understanding the behavior of glycine, other amino acids, and small soluble molecules in solution, as well as the phenomenon of non-classical nucleation more generally.

### Experimental Evidence for Giant Clusters in Undersaturated Aqueous Glycine Solutions

Jawor-Baczynska et al.^2^ studied undersaturated and supersaturated aqueous solutions of glycine (and dl-alanine) by dynamic light scattering (DLS), Brownian microscopy (nanoparticle tracking analysis or NTA), and cryogenic transmission electron microscopy (cryo-TEM). In all cases, they found submicron-sized aggregates in equilibrium with the solutions. These liquid-like nanostructures are reportedly stable, and their size distribution and number concentration depend on solution properties and concentration. Clusters detected by DLS and NTA have a broad size distribution with the mode in the range of 100–300 nm even in glycine solutions at concentrations below 10% of the solid phase solubility limit. A cryo-TEM image apparently shows one of these clusters directly. A simple calculation shows that for near-saturation conditions and in the absence of impurities, these clusters can contain only a minute fraction of all the dissolved glycine (of the order of 0.0001%). Further work shows that similar nanodroplets also exist under supersaturated conditions, and they appear to be important in the pathway to crystallization,^7^ that is, a non-classical crystal nucleation pathway is proposed for aqueous glycine. Repeated filtration and re-crystallization attempts show that the nanodroplets can be removed, but only temporarily. They typically reform in aged samples after a few days or more quickly if stirred. This suggests that they are an equilibrium phase of aqueous glycine.

More recent work includes the detection of glycine clusters with SAXS and NMR.^3^ The presence of nanodroplets in undersaturated solutions is confirmed by SAXS. Moreover, both SAXS and NMR appear to detect the presence of a low concentration of small glycine clusters involving only a few molecules, possibly hydrated glycine pairs.

### Molecular Dynamics Simulations of Aqueous Glycine Solutions

Molecular simulation techniques have also been used to study glycine solutions.^17−22^ These calculations are sensitive to the choice of force field and, especially, atom partial charges, and therefore, it is not surprising that there have been many efforts to obtain suitable atomistic charge sets optimized for aqueous glycine solutions.^21,22^

Studies focused on the investigation of clustering of glycine in aqueous solutions using molecular simulation methods have typically found that while glycine dimers occur frequently, they are not a dominant configuration and have short lifetimes even at relatively high undersaturated concentrations.^17−20^ Therefore, glycine monomers dominate in solution, and only transient clustering between a few glycine molecules is typically reported; giant clusters are absent entirely from these simulations. This agrees with experimental evidence that no more than 10% of glycine molecules in aqueous solution occur in the form of dimers.^3,23^

However, Bushuev et al. reported the cluster size distribution for undersaturated aqueous glycine solutions for cluster sizes >10 molecules.^24^ They found the cluster size distribution to be a quickly decaying function of cluster size, which means that a peak in the cluster size distribution at large cluster sizes is absent. Therefore, in all simulations performed to date, there is no hint of the giant clusters suggested to occur in experiments, although the system sizes used in these simulations are too small to exhibit colloidal-scale objects.

In all the above molecular simulation work, the only glycine species simulated is the zwitterion, which has zero net charge. Charged species of glycine, that is, the anion and cation, which are always present in solution, are absent in these simulations. Since giant cluster formation via the SALR mechanism seems unlikely with only the uncharged zwitterion species present, giant equilibrium clusters are not expected to occur in these simulations. Moreover, it is known that large free-energy barriers can exist between the dispersed state and the clustered state of an SALR cluster fluid.^25^ This means that it is possible that even if giant glycine clusters were stable at equilibrium in such simulations, they might not form from initially dispersed states in a reasonable time. Furthermore, the simulations of undersaturated glycine performed to date have been limited by their size, with typically fewer than 50 zwitterionic glycine molecules. Since it is possible that the critical nucleus size is much larger than this, none of the work performed so far has addressed the questions we seek to address.

To this end, we perform large-scale molecular dynamics (MD) simulations of aqueous glycine looking for signals of giant clustering behavior. We perform simulations with the zwitterion, cation, and anion species and initiate selected simulations from initially clustered states to overcome aggregation barriers. We also add salt (NaCl) to some simulations to gain insights into the potential role of impurities. The remainder of this paper is structured as follows. First, we describe our simulation methods and models. We then examine our simulation results and relate them to experimental data. Finally, we summarize and conclude our work.

## Simulation Methods

**Figure 1:**
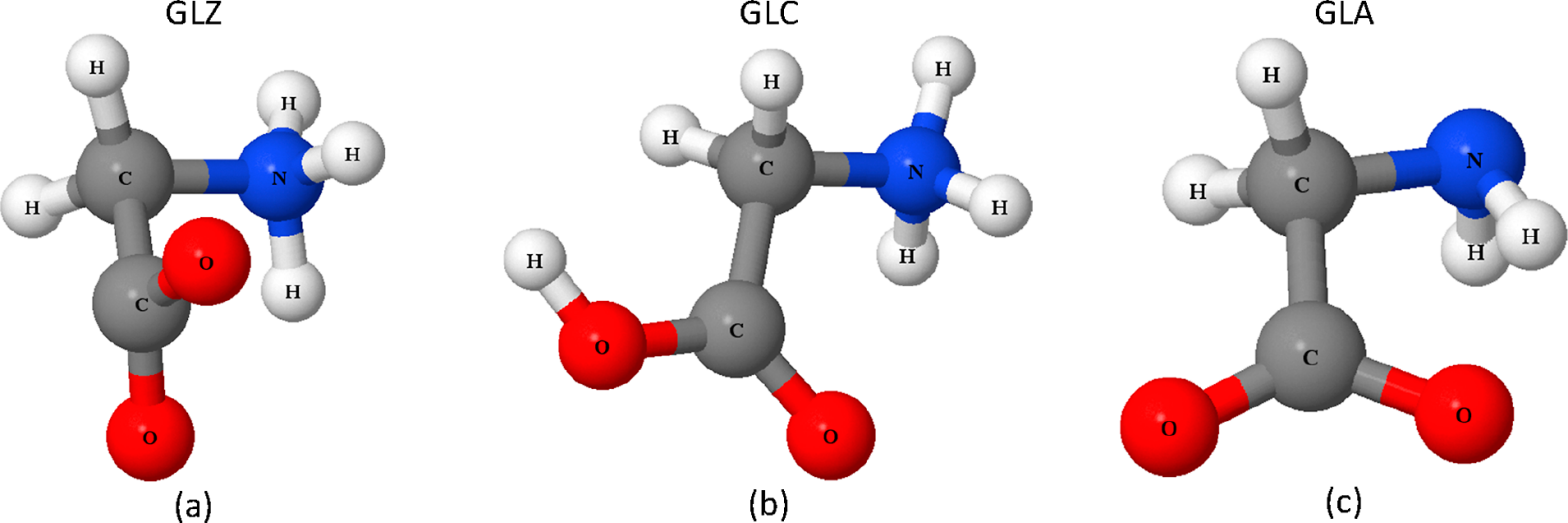
Ball-and-stick models of zwitterion (a), cation (b), and anion (c) glycine molecules.

Gaseous glycine consists mainly of neutral molecules (GLY), but when absorbed into water, glycine predominantly exists in the zwitterionic state (GLZ). The concentration ratio of the zwitterionic (GLZ) to neutral (GLY) forms is roughly 1:10^–5^, while the relative concentration of the anionic (GLA) and cationic (GLC) forms depends strongly on the pH.^26^ At high pH (>9.7), the negatively charged anionic (GLA) form dominates, while at low pH (<2.4), the positively charged cationic (GLC) form dominates. At the isoelectric point, pH ∼ 6, the concentration of GLZ is roughly 1,000 times that of GLC and GLA. It is conceivable that if the giant clusters seen in experiments consist mainly of glycine, they are composed of a mixture of glycine species with an unknown composition. However, considering the low concentration of the neutral form (GLY) at all pH values, we consider it unlikely that it plays any role in cluster formation. Therefore, we require accurate molecular models developed to reproduce sensitive aqueous phase properties for GLZ, GLA, and GLC, as well as water (see Figure 1 for ball-and-stick models of these glycine species).

Considerable effort has been dedicated to creating well-calibrated molecular force fields for organic molecules in the aqueous phase. The 1.14*CM1A charge scheme^27^ is a recent re-calibration of the OPLS-AA force field,^28,29^ with the explicit aim of better reproduction of hydration-free energies. Furthermore, it is currently the only force field available for modeling the glycine cation and anion, GLC and GLA, respectively. We therefore use this force field in our simulations. These models are calibrated with respect to a specific model of water, namely TIP3P, which we therefore also use as our water model.^30^

Atom partial charges, as well as all other OPLS-AA force field parameters (see the Supporting Information), for the 1.14*CM1A partial charge set are easily obtained from the LigParGen website (with three optimization iterations).^31^ We compare them in Table 1 with other partial charge schemes recommended by other authors for the GLZ form. It can be seen that 1.14*CM1A partial charges are slightly more polarized than those recommended by Cheong and Boon^21^ and by Gnanasambandam et al.^22^ after studying different aspects of aqueous glycine behavior. However, those authors recommend the generalized AMBER force field (GAff) or AMBER ff03 values for atomic Lennard-Jones (LJ) interactions instead of the OPLS-AA force field used here, and therefore, there might be some compensation between the LJ and partial charge values for each force field.

**Table 1:** Atom Partial Charges for Several Published Glycine Force Fields, Including Those Used in This Work^a^

	Reference (21) GLZ CNDO	Reference (22) GLZ DNP	this work GLZ 1.14*CM1A	this work GLC CM1A	this work GLA CM1A
N	0.022	–0.127	–0.4549	–0.3735	–0.8552
H	0.164	0.199	0.394	0.3664	0.3183
H	0.208	0.218	0.394	0.3664	0.3183
H	0.199	0.224	0.394	0.3664	
**Subtotal**	**0.593**	**0.514**	**0.7271**	**0.7257**	**–0.2186**
C	–0.021	0.007	–0.2299	–0.1714	–0.0788
H	0.033	0.064	0.1343	0.17055	0.0615
H	0.03	0.061	0.1343	0.17055	0.0615
**Subtotal**	**0.042**	**0.132**	**0.0387**	**0.1697**	**0.0442**
C	0.374	0.483	0.5018	0.4161	0.4162
O	–0.483	–0.578	–0.6338	–0.3234	–0.6209
O	–0.526	–0.552	–0.6338	–0.4205	–0.6209
H				0.4324	
**Subtotal**	**–1.009**	**–1.13**	**–1.2676**	**–0.3115**	**–1.2418**
**Total**	**0**	**–0.001**	**0**	**1**	**–1**

a The “1.14” multiplier for partial charges only applies to neutral molecules.^27,31^ Subtotals are reported for several atomic groups. Note that the slight imbalance reported in ref (22) is likely due to truncation at three decimal places.

There is no prospect of simulating clusters of the size seen in experiments within an MD simulation with atom-scale accuracy. Given the available computing resources, for practical purposes, we chose to perform simulations with a maximum of 450 glycine molecules in various charge states. In experiments,^2,3^ giant clusters are observed at sub-saturated concentrations down to 10 mg/mL, where the solubility limit is 236 mg/mL.^32^ Cluster size is observed to be weakly dependent on concentration, although a general shift to larger clusters is seen on approaching the solubility limit. Clearly, if these clusters are composed mainly of glycine, we expect that they will be more stable at higher glycine concentrations. We therefore perform simulations at concentrations of around half the experimental solubility limit between 125 and 145 mg/mL, where they should form more readily in simulations. For 450 glycine molecules with a range of charge states, this corresponds to 13,500 water molecules.

As it is well known that giant cluster formation can involve high free-energy barriers, which will tend to prevent the formation of giant clusters in simulations initiated from a dispersed state, we initiate some simulations from a pre-formed cluster state. If during a simulation this cluster dissolves, then it is clear that the dispersed state is more stable at this concentration and system size. Likewise, if large clusters are formed in simulations initiated from a dispersed state, then the clustered state is clearly more stable. If, however, both the dispersed and clustered states are apparently stable when initiated from those respective states, no decision can be made on the equilibrium state without recourse to expensive free-energy calculations. Moreover, if a single large cluster persists in the clustered state, we cannot decide from these limited simulations on the equilibrium cluster size or whether a bulk liquid–liquid phase transition would occur in the thermodynamic limit. Given that there are no such bulk liquid–liquid phase transitions observed in experiments, we rule them out on that basis. Therefore, if a single large cluster persists within a simulation, we expect that the equilibrium cluster size is likely to be larger than observed in the simulation.

Our classical MD simulations were carried out using the large-scale atomic/molecular massively parallel simulator (LAMMPS) code^33,34^ accelerated for Intel processors.^35^ The computational work was carried out on the Eddie high-performance computing (HPC) cluster available at the University of Edinburgh. Initial geometries were created using the PACKMOL software package.^36^ For each sample, we performed two independent simulations starting from dispersed and pre-clustered solutions. Table 2 lists concentrations, equilibrium densities, and the number of charged and neutral molecules in each simulation.

**Table 2:** Parameters for the MD Simulations Performed in This Work^a^

simulation	initial state	*C* (mg/mL)	#GLZ	#GLC	#GLA	#Na^+^	#Cl^–^	#H_2_O	density (g/mL)
Pure GLZ	dispersed	143.23	150					4500	1.079
Pure GLZ	clustered	143.1	150					4500	1.078
GLZ + GLC + GLA	dispersed	127.74	150	150	150			13500	1.047
GLZ + GLC + GLA	clustered	127.58	150	150	150			13500	1.046
GLZ + GLC + GLA + NaCl	dispersed	125.59	150	150	150	150	150	13500	1.062
GLZ + GLC + GLA + NaCl	clustered	125.49	150	150	150	150	150	13500	1.061

a # refers to the number of molecules or ions of each type. The density and concentration, *C*, correspond to final states.

Since we did not use a flexible force field for water, all simulations employed a time step of 2.0 fs. Cubic periodic boundary conditions were applied in all directions to mimic bulk liquid samples. Long-range coulombic interactions were evaluated using the particle–particle/particle-mesh solver^33^ using a precision factor of 10^–5^ and a real-space cut-off of 1.2 nm. The short-range interaction cut-off was also set to 1.2 nm. After tight optimization of all initial geometries, MD simulations were performed for between 22 and 28 ns, employing the isothermal–isobaric (*NPT*) ensemble. Simulations were initiated with densities much lower than the ones reported in Table 2, but pressure and temperature equilibration was achieved in every case after a short simulation time in the *NPT* ensemble. The temperature and pressure were controlled using the Martyna–Klein–Tuckerman thermostat and barostat^37^ using temperature and pressure coupling factors of 0.2 and 2.0 ps, respectively.

## Results

**Figure 2:**
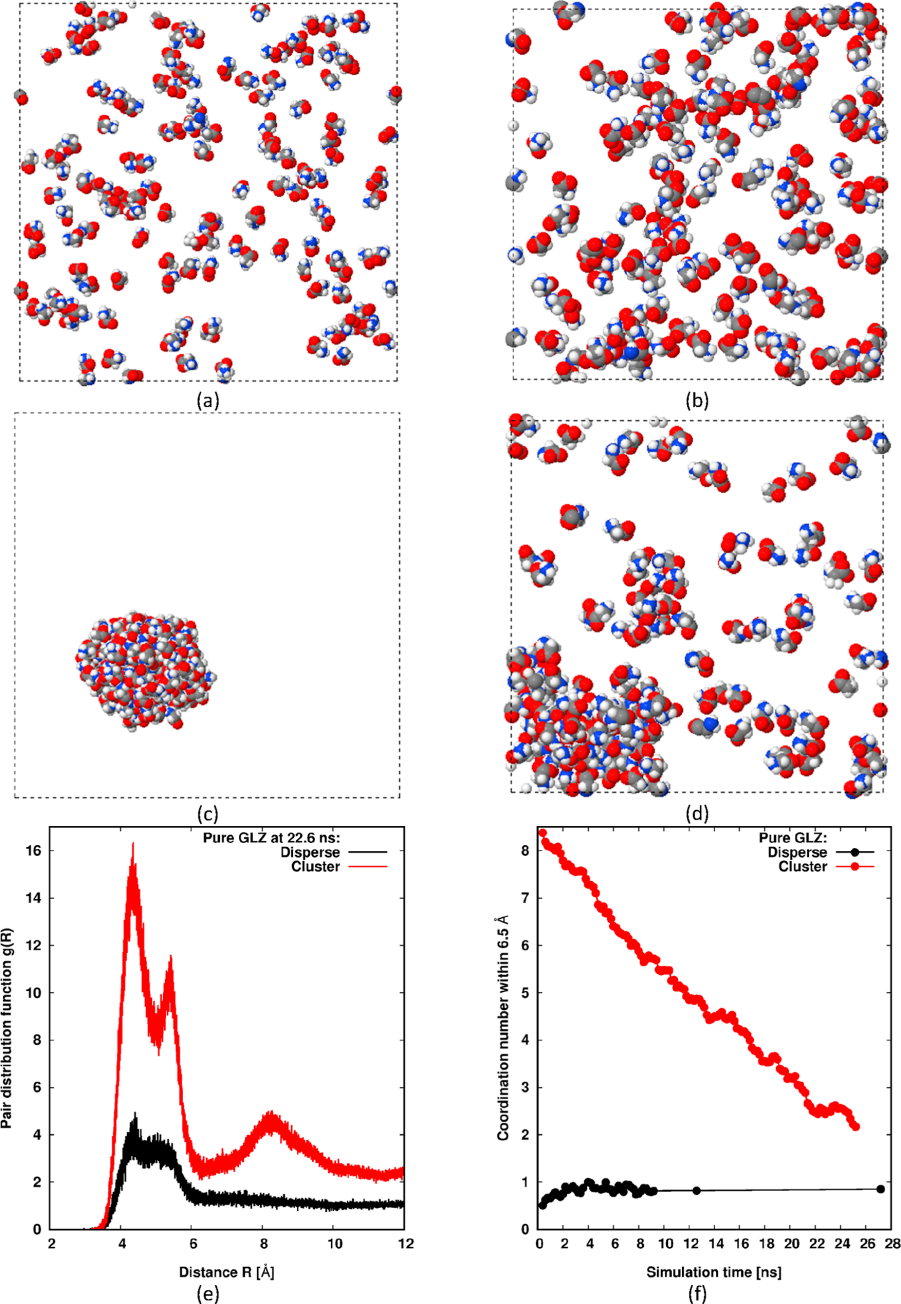
MD simulations of pure GLZ in water. Initial (*t* = 0) dispersed (a) and clustered (c) microstates are shown next to the corresponding near-final (22.0 ns) microstates, (b,d) respectively. The color codes for the snapshots are oxygen—red; nitrogen—blue; carbon—gray; and hydrogen—white. The dashed line denotes the periodic simulation cell boundary. Water molecules are removed for clarity. Lower panel shows near-final (22.6 ns) rdfs for both situations (e) along with the evolution of the coordination number up to 0.65 nm (f).

Beginning with pure GLZ in water, Figure 2a–d show the initial and near-final (22.0 ns) microstates of our *NPT* MD simulation for both situations, that is, where the simulation is started from a dispersed state or from a clustered state. Underneath the snapshots, we plot the near-final radial distribution functions (rdfs), *g*(*R*), at 22.6 ns for these two cases, along with the evolution of the coordination number at 0.65 nm, in Figure 2e,f. The radial distribution functions are based on the nitrogen atom of each molecule (averaged over 0.1 ns), while the coordination number is simply the average number of nitrogen atoms surrounding a central one within 0.65 nm evaluated by integrating the rdf. This choice of cut-off for the coordination number (0.65 nm) essentially highlights the number of nearest neighbors in terms of nitrogen atoms, which corresponds to the first peak in *g*(*R*). For states with large clusters, we expect the rdf to display further peaks at an intermediate range, corresponding to next-nearest neighbors and their neighbors. As separation increases, these intermediate-range peaks will decay to create a slowly decaying plateau in *g*(*R*), which itself will decay to 1 at a long range beyond the diameter of any large cluster in the system. Therefore, intermediate-range bumps in *g*(*R*) are a useful signal of large clusters.

We can clearly see that the system is stable when initiated from a dispersed state; no large cluster is formed even after 27.0 ns. Instead, small clusters and chains of glycine with a slowly decaying population with size are observed in line with the results of Bushuev et al. (although the 2-D presentation likely over-emphasizes the true population of small clusters in the 3-D simulation cell). However, these small clusters are not of interest in this study, in which we seek signals of very large clusters in line with the expectations of an SALR cluster fluid. Such clusters are generally very obvious, with a peak in their size distribution far above 1. As already stated, they typically generate significant bumps in *g*(*R*) at an intermediate range and lead to large values of the coordination number. This behavior is absent in Figure 2e,f for the plots corresponding to initiation from a dispersed state.

Figure 2c,d shows analogous results when initiated with a large pre-formed cluster of GLZ. We see that the cluster quickly disperses into the water. Although a relatively large cluster still remains in the near-final snapshot (Figure 2d), which generates a large bump at an intermediate range in the plot of *g*(*R*) in Figure 2e, it is much smaller than the initial one, and the near-linear evolution of the coordination number (Figure 2f) suggests that it will fully disperse before 40 ns. From this, we conclude that pure GLZ in water is unlikely to form giant clusters spontaneously.

**Figure 3:**
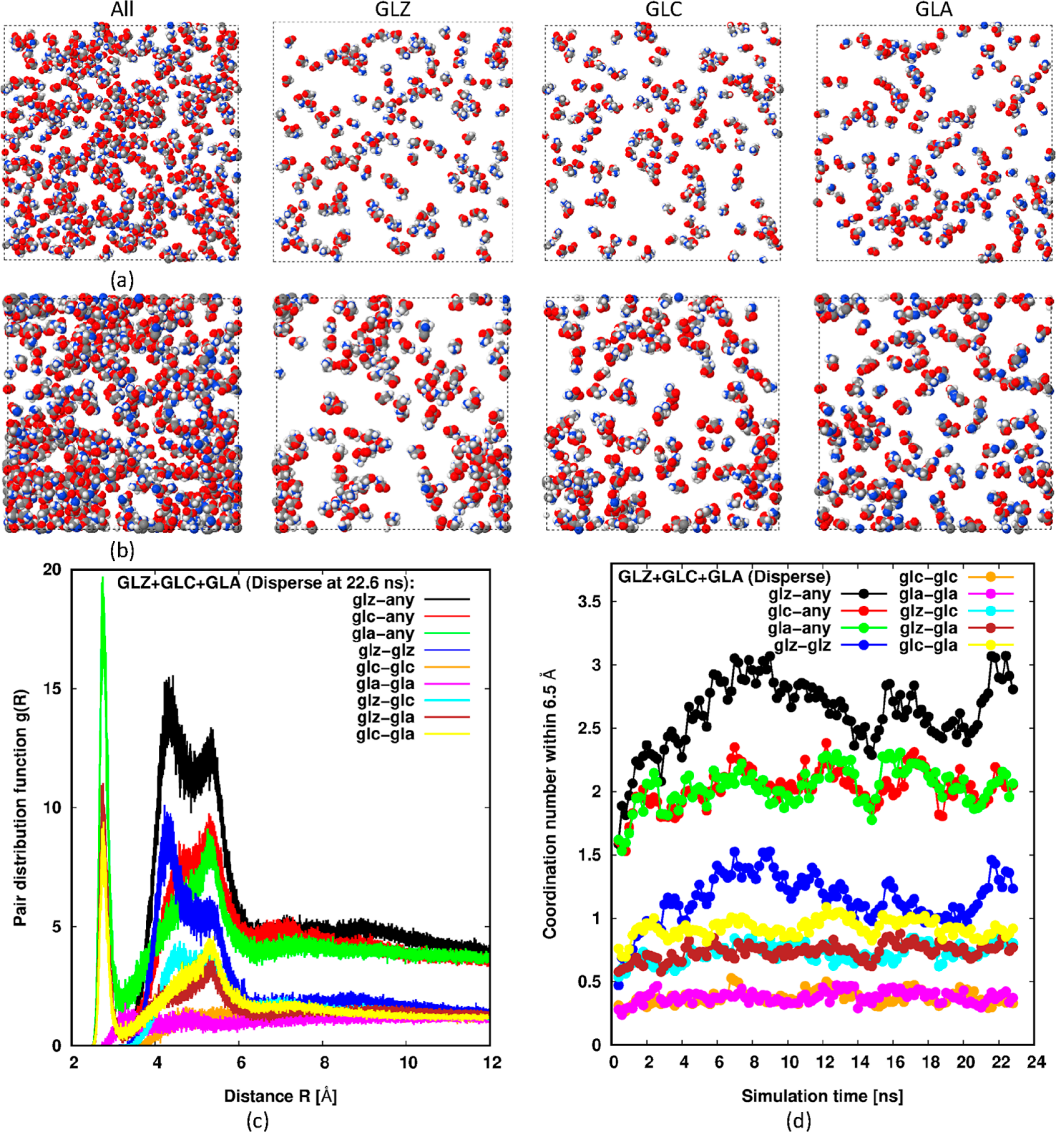
MD simulations of an equimolar mixture of GLZ, GLA, and GLC in water initiated from a dispersed state. Initial (*t* = 0) and near-final (22.0 ns) microstates are shown at the top (a) and middle (b), respectively. The left-most panel in parts (a,b) shows every glycine component, while the other panels only display a specific component; GLZ, GLC, and GLA from left-to-right. The color code is the same as in Figure 2. Lower panel (c) shows the near-final (22.6 ns) partial rdfs along with the evolution of partial coordination numbers up to 0.65 nm in part (d). The rdfs and coordination numbers labeled “x-any” are the sum of the respective partial rdfs and partial coordination numbers. The color codes in these lower two plots are the same.

Figure 3 shows equivalent results for an equimolar mixture of GLZ, GLA, and GLC when initiated from a dispersed state at a similar overall concentration to the pure GLZ case described above. This composition is unrealistic for the *dispersed* state for all values of pH since we would normally expect GLZ to dominate at near-neutral conditions, while GLC is expected to dominate at high pH and GLA is expected to dominate at low pH. However, if the giant clusters seen experimentally are composed mainly of glycine, their composition in terms of the three glycine species is unknown and may well be quite different from the composition observed in the background solution. By including all three species at an equimolar concentration, we aim to detect whether interactions are sufficiently strong between any of them to promote or preserve giant clustering. That is, we expect that if no evidence of clustering is observed even in this ternary mixed case, then clustering is unlikely to occur for any set of mixture concentrations.

Once again, no large clusters are formed during the simulation. No large bumps in *g*(*R*) at the intermediate range are apparent (see Figure 3c), and the coordination numbers for all species remain low in Figure 3d. Among the different components, we see from the rdfs in Figure 3c, and especially the coordination numbers in Figure 3d that the strongest effective interactions are mutual interactions between GLZ molecules. Perhaps surprisingly, they appear to be as intense as the effective interactions between GLC and GLA, which have net opposite charges, but occur over a wider range of separations (see Figure 3c) resulting in a greater net attraction and significantly more nearest neighbors in Figure 3d. This suggests that water is able to effectively screen the strong interaction between oppositely charged glycine ions. Another feature is observed in *g*(*R*) at short range, ∼0.28 nm, for the plots involving the anion GLA. This sharp peak is caused by the reduction of charge on the amine group of the anion, which allows nitrogen atoms to approach more closely.

**Figure 4:**
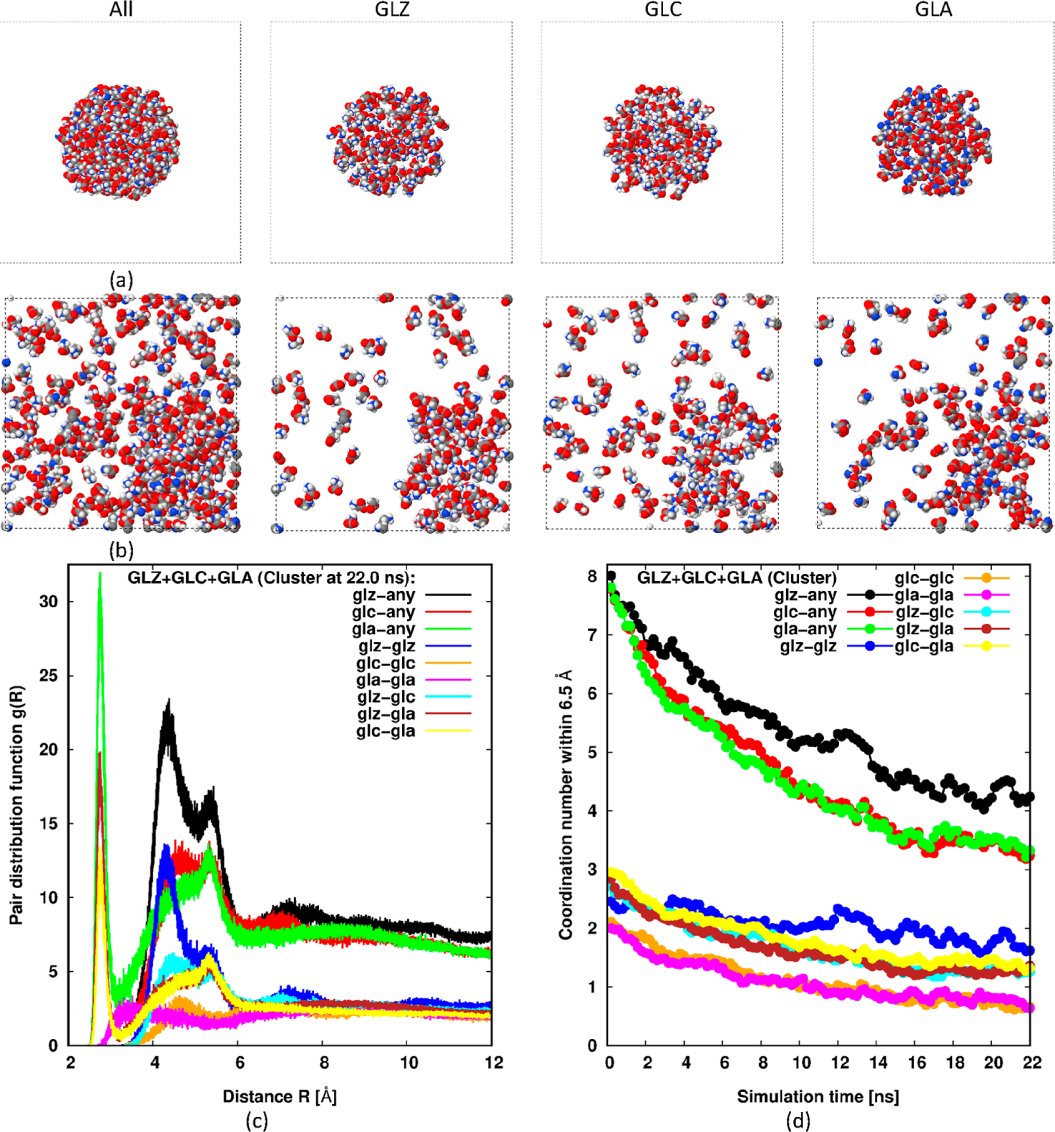
Snapshots and data analogous to Figure 3, except that simulations are initiated from a clustered state.

Figure 4 shows results analogous to Figure 3, except now the simulation is initiated from a clustered state. As we have just shown that large clusters of pure GLZ are unlikely to be stable, and the strongest interactions are expected to occur between GLZ molecules, we do not expect this large mixed cluster to be stable either, and this is borne out in the simulation. Indeed, we see that the initial large cluster gradually decays, and it continues to disperse at the end of the simulation. Once again, from the coordination number plots (Figure 4d), we see that the strongest interaction is the mutual one between GLZ molecules, and there is no special affinity between GLC and GLA. While this suggests the cluster should decay more quickly than in the case of pure GLZ in Figure 2, this is not the case. Indeed, it is not obvious from this simulation whether the final state will be fully dispersed or whether a small cluster will remain at equilibrium. However, given that the coordination number for “GLZ-any” in the dispersed system in Figure 3d attains values higher than 3.0 near the end of the simulation, while the corresponding coordination number for the clustered system, in Figure 4d, attains values close to 4.0 by the end of the simulation and appears to be decreasing further, it seems likely that the remaining cluster will probably also disperse given sufficient simulation time.

Moreover, the concentration of glycine molecules in the dispersed phase outside of the cluster becomes quite high toward the end of the simulation, which is at odds with the experimental results, which show that giant clusters persist even at quite low glycine concentrations. Of course, it remains a possibility that our simulations are still too small and below the threshold required to sustain a giant glycine cluster, that is, the real critical nucleus is larger than 450 molecules. Nevertheless, we conclude from this result that large glycine clusters are probably unstable in water under these conditions. Considering that this concentration is far higher than that observed experimentally for some giant clusters and that our composition is chosen to reveal potential signals of clustering between any of the three components, we do not expect the experimentally observed clusters to be formed of glycine alone.

If the giant clusters seen experimentally do not consist of pure glycine, they might alternatively be nucleated around impurities. In experiments, the pH of glycine solutions is tailored by adding strong acids or bases, such as HCl and NaOH. Therefore, salt ions are one obvious impurity that might play a role in giant cluster formation. To examine this possibility, we repeated the above simulations corresponding to Figures 3 and 4 but with added salt ions.

**Figure 5:**
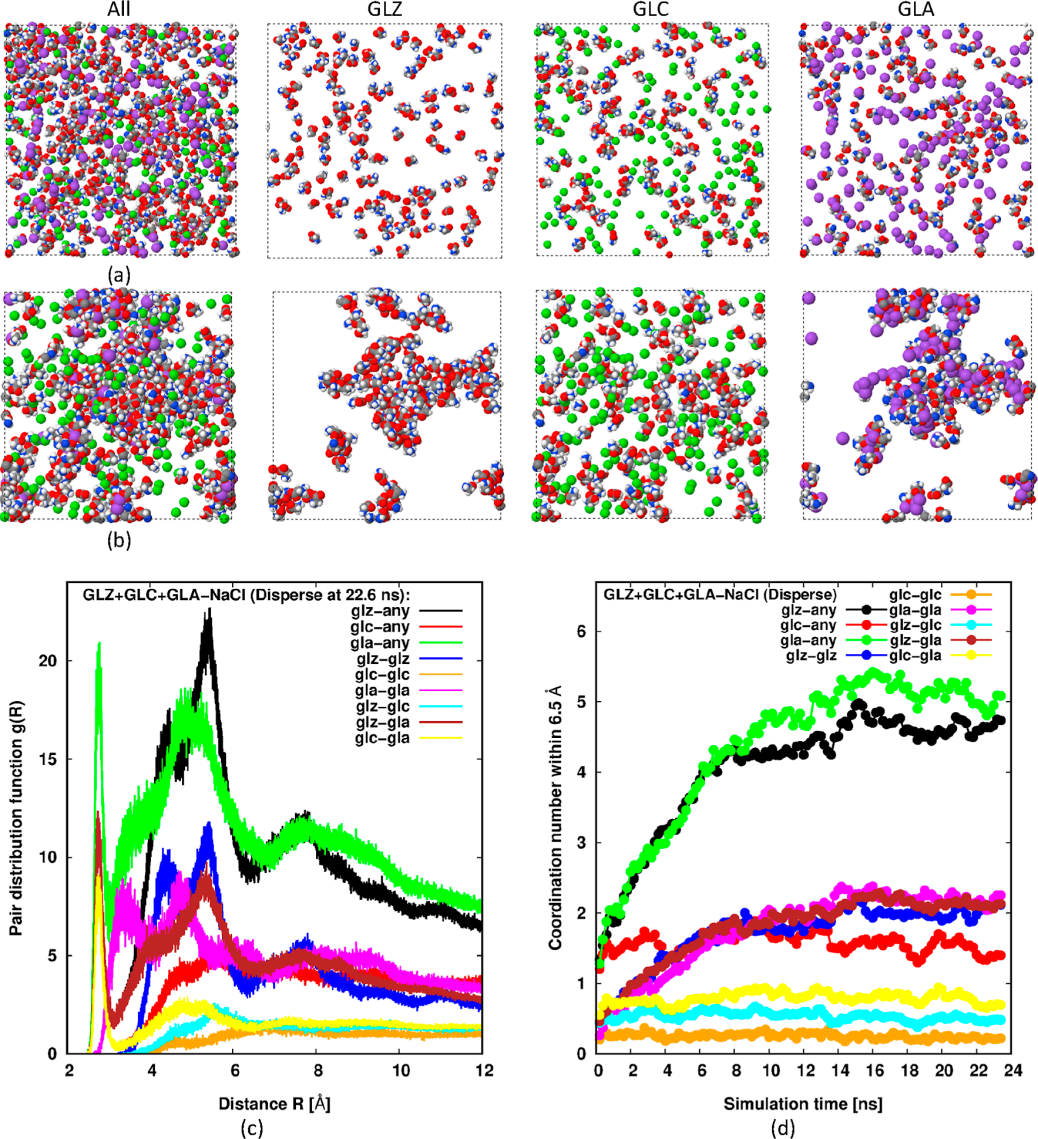
Snapshots and data analogous to Figure 3, except an equimolar amount of salt ions are added. The GLC snapshot also shows Cl^–^, while the GLA snapshot also shows Na^+^.

Figure 5 shows data from the simulation resulting from adding an equimolar mixture of salt ions, Na^+^ and Cl^–^, to an equimolar mixture of GLZ, GLC, and GLA. Essentially, the simulation now contains 150 molecules or ions of each type, along with 13,500 water molecules (see Table 2). By the end of the simulation, we see from Figure 5b that several fractal-like clusters of GLZ + GLA + Na^+^ have formed. The cation, GLC, and Cl^–^ remain in a dispersed state. Cluster growth is tracked by the coordination number plots in Figure 5d, while Figure 5c confirms their presence at the end of the simulation by displaying a large bump in *g*(*R*) at an intermediate range.

The reason for this preferential clustering involving GLA or GLZ and Na^+^ is not obvious. One possibility can be deduced from Table 1, which shows the atomic partial charges involved. We see that both GLZ and GLA feature strong charge density at the anionic terminus (1.27 e on the OO^–^ group), where the pair of oxygen atoms shield the positively charged carbon to which they are bonded to some extent. Comparison with the cationic terminus of GLZ and GLC, NH_3_^+^, shows that it has a weaker charge density (−0.73 e on the NH_3_^+^ group), where the hydrogen atoms are too small to shield the nitrogen atom to which they are bonded. Presumably, then, water is unable to effectively screen a small ion like Na^+^ against this strongly charged OO^–^ region of both GLA and GLZ, while it can screen Cl^–^ against the more weakly charged NH_3_^+^ region of GLC and GLZ. Another possibility for this preference to cluster around sodium ions is that there might be a hydration asymmetry between the sodium and chlorine ions.^40^

**Figure 6:**
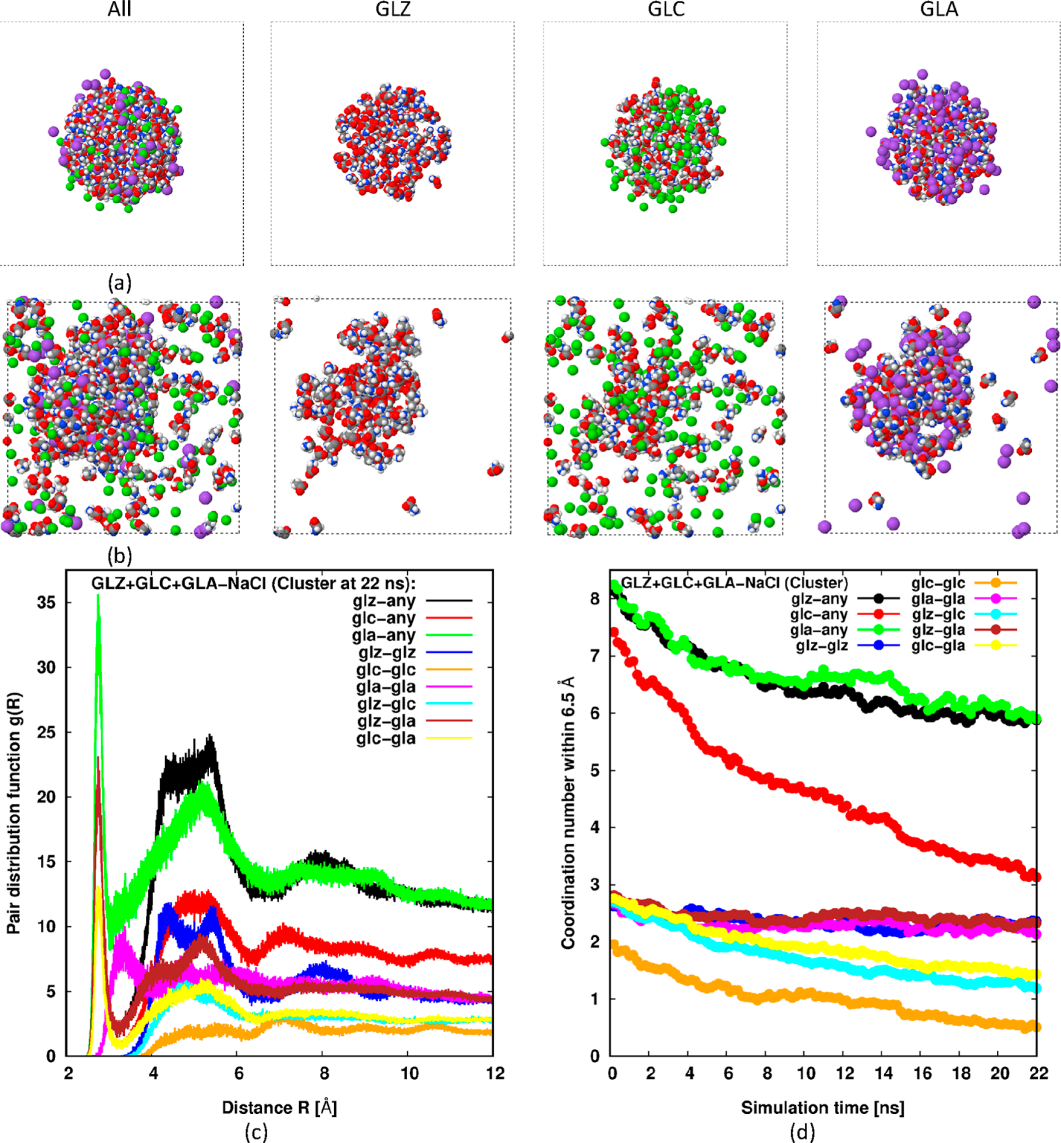
Snapshots and data analogous to Figure 4, except an equimolar amount of salt ions are added. The GLC snapshot also shows Cl^–^, while the GLA snapshot also shows Na^+^.

These clusters are composed of oppositely charged species, GLA + Na^+^, plus the zwitterion GLZ. Given that they form spontaneously from a dispersed solution, we can expect that they will also persist when the simulation is initiated from a clustered state. And indeed, this is the case. Figure 6 shows the analogous data for this situation. We see from Figure 6b that a single large cluster of GLZ + GLA + Na^+^ remains in the simulation, while the GLC and Cl^–^ again disperse. The coordination plots in Figure 6d, which show only slow decay toward the end of the simulation, confirm that the cluster is stable after the GLC and Cl^–^ disperse, while Figure 6c again displays a significant bump in *g*(*R*) at the intermediate range. Unfortunately, we cannot determine the equilibrium size of the cluster under these conditions since our simulations are likely to be limited by finite size effects. That is, we cannot know from this single simulation whether, for a much larger simulation, a single cluster, or multiple clusters with a similar size to the one here, is the equilibrium state.

## Discussion and Conclusions

Our MD simulations show that, at least for this model of glycine in water at undersaturated conditions, pure glycine does not show any tendency to form giant clusters. This also applies when all species of glycine, GLZ, GLA, and GLC, are considered. This is our main result, which has not been reported before.

Even though the composition of the clusters observed in experiments is unknown, by performing simulations with a high concentration of each species initiated from a clustered state, it is apparent that none of the interactions between any of the species is sufficiently strong to maintain a clustered state. Therefore, we find little support for the hypothesis that experimentally observed giant clusters in aqueous glycine solutions below the solubility limit are composed mainly of glycine and are formed without the action of any impurities.

However, we do find large stable clusters involving glycine when small positive ions, corresponding to salt or a strong base, are added to the solutions. This finding supports the view that the giant clusters observed in experiments are probably triggered by the presence of impurities, especially charged impurities that form complex clusters with GLZ and GLA.

Set against this view is the fact that giant clusters are observed even in aqueous glycine solutions that have been through several rounds of re-crystallization and filtration that aim to remove such impurities.^2,3^ Therefore, it is doubtful that there are sufficient levels of such salt impurities remaining in those experiments to trigger any significant clustering behavior. Moreover, when a small amount of salt is deliberately added to aqueous glycine, there is no obvious change observed in the giant clusters.^3^ This negative experimental result might indicate that despite our best efforts, our force fields are inadequate. Alternatively, the clustering observed with added salt in our simulations might be of a different kind to the nanodroplets observed in experiments.

Ultimately, we still do not have a clear picture of the identity and formation mechanism of the giant clusters observed experimentally, although it seems unlikely that they are formed of pure glycine. Of course, there are also several problems with our simulation methods that might prevent large clusters from forming, and therefore, our conclusion must remain somewhat qualified. Most obviously, our results depend sensitively on the force fields used. Although we have tried to select a good force field based on the 1.14*CM1A protocol, which has been optimized to reproduce hydration-free energies, this model might still be inadequate to realistically reproduce glycine aggregation. In addition, our simulations remain very small compared to the cluster size observed experimentally. Therefore, it is possible that we have not exceeded the critical cluster size for giant cluster nucleation.

Despite these potential issues, there are good reasons for thinking that the results here are a fair reflection of reality and therefore that the giant clusters observed in aqueous glycine experiments are probably not composed purely or mainly of glycine. One strong argument against them being composed primarily of glycine is that the concentration of giant clusters in the experiments is very low. Indeed, we estimate that even close to the saturation limit, if the nanodroplets are composed mainly of glycine, less than one in a million glycine molecules is involved in their formation. This is a tiny proportion that appears to hold for a wide range of glycine concentrations.

This behavior cannot be explained by the SALR mechanism for giant cluster formation, or indeed by liquid droplet nucleation in the process of bulk phase separation, if the clusters are formed primarily by glycine. In either case, we would expect to see the proportion of molecules in the liquid-like phase grow very quickly using arguments similar to the Lever rule once the critical cluster concentration or dew point concentration is exceeded. However, this has not been observed in any experiment. These observations hint at cluster formation controlled by impurities, in agreement with the simulations we present here. And yet, strenuous efforts have been made to remove all such impurities in experiments.

A potential solution to this apparent contradiction is that in aqueous solutions, glycine undergoes several reactions spontaneously, forming two main kinds of products. On the one hand, aqueous glycine undergoes a dehydration reaction to form the short peptide diglycine. This dehydration reaction is relatively slow, and, of course, the reaction equilibrium is biased heavily toward reactants. Nevertheless, at equilibrium, we expect the concentration of dipeptide to be about 1,000th that of glycine,^38^ similar to the glycine cation and anion, GLC and GLA, under neutral conditions near the isoelectric point. In turn, further dehydration reactions generate a hierarchy of longer glycine peptides, each with a decreasing concentration at equilibrium with aqueous glycine.^39^

Although we do not expect the equilibrium concentrations of these peptide reaction products to exceed their solubility limits^40^ and they are not observed to precipitate in experiments, they might nevertheless aggregate to form liquid-like clusters in solution. Indeed, simulations involving aqueous pentaglycine suggest that it spontaneously forms large clusters in solution.^41^ However, these simulations are performed at pentaglycine concentrations far higher than the concentration of pentaglycine expected in equilibrium with aqueous glycine. Therefore, it is unclear whether pentaglycine is a solution to our problem. Unfortunately, clustering involving smaller glycine peptides has not been investigated in this way.

Alternatively, a more interesting reaction product of aqueous glycine is diketopiperazine. This molecule is essentially the cyclic version of diglycine and is formed through two dehydration reactions. Its equilibrium concentration relative to diglycine in aqueous glycine solutions is not reported, although the formation of diketopiperazine might be favored in the presence of a silica catalyst.^42^

Very interestingly, diketopiperazine is a powerful small-molecule gelling agent,^43^ and its solubility in water is less than 10% that of glycine, being around 1.5% by weight under standard conditions.^44^ Indeed, a great deal of research in recent years has focused on diketopiperazine and its derivatives for a wide range of applications,^45^ especially biomedical ones such as cancer treatment, for which it is thought to have significant advantages over linear peptides.^46^ The gelation property of diketopiperazines has been extensively studied and persists at a low concentration. It is caused by the tendency of diketopiperazines to form long hydrogen-bonded chains in solution owing to the possibility for each molecule to be involved in four hydrogen bonds, that is, two per molecule in the chain.^43,45^ Side chains derived from amino acids other than glycine can alter this chain-like structure, leading to planar, fibrillar, and other mesostructures in solution.^47^ However, there appears to be no research into the physical structures formed by diketopiperazines at concentrations below the gelation limit. Therefore, it is unclear whether diketopiperazine spontaneously forms giant clusters in aqueous solutions below the gelation limit.

Nevertheless, this behavior might explain some of the experimental observations in aqueous glycine solutions in several respects. First, we know that only a tiny fraction of the molecules in aqueous glycine solutions are involved in the formation of the clusters observed, and this indicates low-concentration impurities with strong short-ranged attractions. Diketopiperazine will form at low concentrations in such experiments, and its mutual interaction through a network of hydrogen bonds suggests the possibility of a strong short-ranged attraction. Second, its formation rate is relatively slow, and thus, even if removed by re-crystallization and washing, it will inevitably re-appear in solution after a significant delay. Again, this agrees with observations that giant clusters only reform in glycine solutions several hours to days after re-crystallization attempts. Finally, silica, a component of glass used for containers and stirring rods in experiments, might act as a weak catalyst for the formation of diketopiperazine from glycine.

If the large clusters observed in aqueous glycine experiments are indeed formed primarily of diketopiperazine or other oligoglycines, then we can expect that they will also absorb significant amounts of glycine, the dominant solute, from solution. Therefore, these nanodroplets might well consist of a mixture of components, including glycine and its many reaction products in solution. Furthermore, small oligoglycines may be difficult to separate effectively from aqueous glycine using conventional separation methods. Even for crystallization, this may be challenging due to the potential formation of multicomponent solid phases, such as crystalline solid solutions, which have been observed for some amino acids.^48^

Therefore, our main recommendation from this work is that the focus of this investigation should shift to the study of reaction products of aqueous glycine, that is, small oligoglycines and especially diketopiperazine. Although these reaction products occur at relatively low concentrations in aqueous glycine solutions, some of them, like diketopiperazine, might exhibit sufficiently strong short-ranged attractions due to hydrogen bonding or cross-interactions with glycine to trigger aggregation. Moreover, because diketopiperazine is a secondary amine with two amine sites, the long-ranged repulsion required for stabilization of giant clusters via the SALR mechanism is also apparent through protonation of a proportion of the secondary amine sites.

## Supporting Information Available

Full details of the force field used for each glycine component (ZIP)

All files used to perform these simulations are available at https://doi.org/10.7488/ds/3477.

## References

[ref1] Elsila J. E.; Glavin D. P.; Dworkin J. P. Cometary glycine detected in samples returned by Startdust. Meteorit. Planet. Sci. 2009, 44, 1323–1330. 10.1111/j.1945-5100.2009.tb01224.x.

[ref2] Jawor-Baczynska A.; Moore B. D.; Lee H. S.; McCormick A. V.; Sefcik J. Population and size distribution of solute-rich mesospecies within mesostructured aqueous amino acid solutions. Faraday Discuss. 2013, 167, 425–440. 10.1039/c3fd00066d. 24640504

[ref3] Zimbitas G.; Jawor-Baczynska A.; Vesga M. J.; Javid N.; Moore B. D.; Parkinson J.; Sefcik J. Investigation of molecular and mesoscale clusters in undersaturated glycine aqueous solutions. Colloids Surf., A 2019, 579, 123633 10.1016/j.colsurfa.2019.123633.

[ref4] Karthika S.; Radhakrishnan T. K.; Kalaichelvi P. A review of classical and non-classical nucleation theories. Cryst. Growth Des. 2016, 16, 6663–6681. 10.1021/acs.cgd.6b00794.

[ref5] Bonnett P. E.; Carpenter K. J.; Dawson S.; Davey R. J. Solution crystallisation via a submerged liquid-liquid phase boundary: oiling out. Chem. Commun. 2003, 6, 698–699. 10.1039/b212062c. 12703779

[ref6] Zhang F. Nonclassical nucleation pathways in protein crystallization. J. Phys.: Condens. Matter 2017, 29, 443002 10.1088/1361-648x/aa8253. 28984274

[ref7] Jawor-Baczynska A.; Sefcik J.; Moore B. D. 250 nm glycine-rich nanodroplets are formed on dissolution of glycine crystals but are too small to provide productive nucleation. Cryst. Growth Des. 2013, 13, 470–478. 10.1021/cg300150u.

[ref8] Gowayed O. Y.; Moosa T.; Moratos A. M.; Hua T.; Arnold S.; Garetz B. A. Dynamic light scattering study of a laser-induced phase-separated droplet of aqueous glycine. J. Phys. Chem. B 2021, 125, 7828–7839. 10.1021/acs.jpcb.1c02620. 34259002

[ref9] Alexander A. J.; Camp P. J. Non-photochemical laser-induced nucleation. J. Chem. Phys. 2019, 150, 040901 10.1063/1.5079328. 30709291

[ref10] Javid N.; Kendall T.; Burns I. S.; Sefcik J. Filtration suppresses laser-induced nucleation of glycine in aqueous solutions. Cryst. Growth Des. 2016, 16, 4196–4202. 10.1021/acs.cgd.6b00046.

[ref11] Dinsmore A.; Dubin P.; Grason G. Clustering in complex fluids. J. Phys. Chem. B 2011, 115, 7173–7174. 10.1021/jp202724b. 21631115

[ref12] Meilhac N.; Destainville N. Clusters of proteins in biomembranes: Insights into the roles of interaction potential shapes and of protein diversity. J. Phys. Chem. B 2011, 115, 7190–7199. 10.1021/jp1099865. 21528886

[ref13] Cardinaux F.; Zaccarelli E.; Stradner A.; Bucciarelli S.; Farago B.; Egelhaaf S. U.; Sciortino F.; Schurtenberger P. Cluster-driven dynamical arrest in concentrated lysozyme solutions. J. Phys. Chem. B 2011, 115, 7227–7237. 10.1021/jp112180p. 21528887

[ref14] Viet M. H.; Ngo S. T.; Lam N. S.; Li M. S. Inhibition of aggregation of amyloid peptides by peta-sheet breaker peptides and their binding affinity. J. Phys. Chem. B 2011, 115, 7433–7446. 10.1021/jp1116728. 21563780

[ref15] Sweatman M. B.; Lue L. The giant SALR cluster fluid: A review. Adv. Theory Simul. 2019, 2, 1900025 10.1002/adts.201900025.

[ref16] Sweatman M. B.; Fartaria R.; Lue L. Cluster formation in fluids with competing short-range and long-range interactions. J. Chem. Phys. 2014, 140, 124508 10.1063/1.4869109. 24697460

[ref17] Campo M. G. Molecular dynamics simulations of glycine zwitterion in aqueous solution. J. Chem. Phys. 2006, 125, 114511 10.1063/1.2352756. 16999494

[ref18] Hamad S.; Hughes C. E.; Catlow C. R. A.; Harris K. D. M. Clustering of glycine molecules in aqueous solution studied by molecular dynamics simulation. J. Phys. Chem. B 2008, 112, 7280–7288. 10.1021/jp711271z. 18503273

[ref19] Yani Y.; Chow P. S.; Tan R. B. H. Glycine open dimers in solution: New insights into α-glycine nucleation and growth. Cryst. Growth Des. 2012, 12, 4771–4778. 10.1021/cg300452n.

[ref20] Di Gioacchino M.; Ricci M. A.; Imberti S.; Holzmann N.; Bruni F. Hydration and aggregation of a simple amino acid: The case of glycine. J. Mol. Liq. 2020, 301, 112407 10.1016/j.molliq.2019.112407.

[ref21] Cheong D. W.; Boon Y. D. Comparative study of force fields for molecular dynamics simulations of α-glycine crystal growth from solution. Cryst. Growth Des. 2010, 10, 5146–5158. 10.1021/cg100906s.

[ref22] Gnanasambandam S.; Hu Z.; Jiang J.; Rajagopalan R. Force field for molecular dynamics studies of glycine/water mixtures in crystal/solution environments. J. Phys. Chem. B 2009, 113, 752–758. 10.1021/jp802949u. 19115812

[ref23] Huang J.; Stringfellow T. C.; Yu L. Glycine Exists Mainly as Monomers, Not Dimers, in Supersaturated Aqueous Solutions: Implications for Understanding Its Crystallization and Polymorphism. J. Am. Chem. Soc. 2008, 130, 13973–13980. 10.1021/ja804836d. 18816054

[ref24] Bushuev Y. G.; Davletbaeva S. V.; Koifman O. I. Molecular dynamics simulations of aqueous glycine solutions. CrystEngComm 2017, 19, 7197–7206. 10.1039/c7ce01271c.

[ref25] Sweatman M. B. Giant SALR cluster reproduction, with implications for their chemical evolution. Mol. Phys. 2018, 116, 1945–1952. 10.1080/00268976.2017.1406164.

[ref26] Wada G.; Tamura E.; Okina M.; Nakamura M. On the ratio of zwitterion form to uncharged form of glycine at equilibrium in various aqueous media. Bull. Chem. Soc. Jpn. 1982, 55, 3064–3067. 10.1246/bcsj.55.3064.

[ref27] Dodda L. S.; de Vaca I.; Tirado-Rives J.; Jorgensen W. L. LigParGen web server: an automatic OPLS-AA parameter generator for organic ligands. Nucleic Acids Res. 2017, 45, W331–W336. 10.1093/nar/gkx312. 28444340 PMC5793816

[ref28] Jorgensen W. L.; Maxwell D. S.; Tirado-Rives J. Development and testing of the OPLS all-atom force field on conformational energetics and properties of organic liquids. J. Am. Chem. Soc. 1996, 118, 11225–11236. 10.1021/ja9621760.

[ref29] Jorgensen W. L.; Tirado-Rives J. Potential energy functions for atomic-level simulations of water and organic and biomolecular systems. Proc. Natl. Acad. Sci. 2005, 102, 6665–6670. 10.1073/pnas.0408037102. 15870211 PMC1100738

[ref30] Price D. J.; Brooks C. L. A modified TIP3P water potential for simulation with Ewald summation. J. Chem. Phys. 2004, 121, 10096 10.1063/1.1808117. 15549884

[ref31] http://zarbi.chem.yale.edu/ligpargen/ (accessed on March 08, 2022).

[ref32] Ferreira L. A.; Macedo E. A.; Pinho S. P. Effect of KCl and Na2SO4 on the Solubility of Glycine and DL-Alanine in Water at 298.15 K. Ind. Eng. Chem. Res. 2005, 44, 8892–8898. 10.1021/ie050613q.

[ref33] Plimpton S. Fast parallel algorithms for short-range molecular dynamics. J. Comput. Phys. 1995, 117, 1–19. 10.1006/jcph.1995.1039.

[ref34] https://www.lammps.org/ (accessed on March 08, 2022).

[ref35] Brown W.; Carrillo J.-M. Y.; Gavhane N.; Thakkar F. M.; Plimpton S. J. Optimising legacy molecular dynamics software with directive-based offload. Comput. Phys. Commun. 2015, 195, 95–101. 10.1016/j.cpc.2015.05.004.

[ref36] Martínez L.; Andrade R.; Birgin E. G.; Martínez J. M. PACKMOL: a package for building initial configurations for molecular dynamics simulations. J. Comput. Chem. 2009, 30, 2157–2164. 10.1002/jcc.21224. 19229944

[ref37] Tuckerman M. E.; Alejandre J.; López-Rendón R.; Jochim A. L.; Martyna G. J. A Liouville-operator derived measure-preserving integrator for molecular dynamics simulations in the isothermal-isobaric ensemble. J. Phys. A: Math. Gen. 2006, 39, 5629–5651. 10.1088/0305-4470/39/19/s18.

[ref38] Mancinelli R.; Botti A.; Bruni F.; Ricci M. A.; Soper A. K. Hydration of Sodium, Potassium, and Chloride Ions in Solution and the Concept of Structure Maker/Breaker. J. Phys. Chem. B 2007, 111, 13570–13577. 10.1021/jp075913v. 17988114

[ref39] Martin R. B. Free energies and equilibria of peptide bond hydrolysis and formation. Biopolymers 1998, 45, 351–353. 10.1002/(sici)1097-0282(19980415)45:5<351::aid-bip3>3.0.co;2-k.

[ref40] Lu J.; Wang X.-J.; Yang X.; Ching C.-B. Solubilities of glycine and its oligopeptides in aqueous solutions. J. Chem. Eng. Data 2006, 51, 1593–1596. 10.1021/je0600754.

[ref41] Karandur D.; Wong K.-Y.; Pettitt B. M. Solubility and aggregation of gly5 in water. J. Phys. Chem. B 2014, 118, 9565–9572. 10.1021/jp503358n. 25019618 PMC4136715

[ref42] Bujdák J.; Rode B. M. Silica, alumina and clay catalysed peptide bond formation: Enhanced efficiency of alumina catalyst. Orig. Life Evol. Biosph. 1999, 29, 451–461. 10.1023/a:1006524703513. 10573687

[ref43] Scarel M.; Marchesan S. Diketopiperazine gels: New horizons from the self-assembly of cyclic dipeptides. Molecules 2021, 26, 3376 10.3390/molecules26113376. 34204905 PMC8199760

[ref44] Suzuki K.; Tsuchiya M.; Kadono H. Effect of pressure on the solubility of diketopiperazine in water. Bull. Chem. Soc. Jpn. 1970, 43, 3083–3086. 10.1246/bcsj.43.3083.

[ref45] Manchineella S.; Govindaraju T. Molecular self-assembly of cyclic dipeptide derivatives and their applications. ChemPlusChem 2017, 82, 88–106. 10.1002/cplu.201600450. 31961506

[ref46] Bojarska J.; Mieczkowski A.; Ziora Z. M.; Skwarczynski M.; Toth I.; Shalash A. O.; Parang K.; El-Mowafi S. A.; Mohammed E. H. M.; Elnagdy S.; et al. Cyclic dipeptides: The biological and structural landscape with special focus on the anti-cancer proline-based scaffold. Biomolecules 2021, 11, 1515 10.3390/biom11101515. 34680148 PMC8533947

[ref47] Joshi K. B.; Verma S. Participation of aromatic side chains in diketopiperazine ensembles. Tetrahedron Lett. 2008, 49, 4231–4234. 10.1016/j.tetlet.2008.04.156.

[ref48] Raza S. A.; Schacht U.; Svoboda V.; Edwards D. P.; Florence A. J.; Pulham C. R.; Sefcik J.; Oswald I. D. H. Rapid continuous antisolvent crystallization of multicomponent systems. Cryst. Growth Des. 2017, 18, 210–218. 10.1021/acs.cgd.7b01105.

