# Supplementary material for: Molecular Dynamics Investigation of Clustering in Aqueous Glycine Solutions
Source: J Phys Chem B. 2022 Jun 21;126(25):4711–22. doi: 10.1021/acs.jpcb.2c01975 (PMC9251761; doi:10.1021/acs.jpcb.2c01975)
Supplement: Supplementary file 1 — jp2c01975_si_001.zip [file jp2c01975_si_001.zip › GLA.pdf]

This XML file does not appear to have any style information associated with it. The document tree is shown below.


```
<ForceField>
  <AtomTypes>
    <Type name="opls_804" class="O804" element="O" mass="15.999000"/>
    <Type name="opls_808" class="H808" element="H" mass="1.008000"/>
    <Type name="opls_807" class="H807" element="H" mass="1.008000"/>
    <Type name="opls_801" class="C801" element="C" mass="12.011000"/>
    <Type name="opls_806" class="H806" element="H" mass="1.008000"/>
    <Type name="opls_802" class="C802" element="C" mass="12.011000"/>
    <Type name="opls_800" class="N800" element="N" mass="14.007000"/>
    <Type name="opls_803" class="O803" element="O" mass="15.999000"/>
    <Type name="opls_805" class="H805" element="H" mass="1.008000"/>
  </AtomTypes>
  <Residues>
    <Residue name="UNK">
      <Atom name="N00" type="opls_800"/>
      <Atom name="C01" type="opls_801"/>
      <Atom name="C02" type="opls_802"/>
      <Atom name="O03" type="opls_803"/>
      <Atom name="O04" type="opls_804"/>
      <Atom name="H05" type="opls_805"/>
      <Atom name="H06" type="opls_806"/>
      <Atom name="H07" type="opls_807"/>
      <Atom name="H08" type="opls_808"/>
      <Bond from="0" to="1"/>
      <Bond from="1" to="2"/>
      <Bond from="2" to="3"/>
      <Bond from="2" to="4"/>
      <Bond from="0" to="5"/>
      <Bond from="0" to="6"/>
      <Bond from="1" to="7"/>
      <Bond from="1" to="8"/>
    </Residue>
  </Residues>
  <HarmonicBondForce>
    <Bond class1="C801" class2="N800" length="0.144800" k="319657.600000"/>
    <Bond class1="C802" class2="C801" length="0.152200" k="265265.600000"/>
    <Bond class1="O803" class2="C802" length="0.125000" k="548940.800000"/>
    <Bond class1="O804" class2="C802" length="0.125000" k="548940.800000"/>
    <Bond class1="H805" class2="N800" length="0.101000" k="363171.200000"/>
    <Bond class1="H806" class2="N800" length="0.101000" k="363171.200000"/>
    <Bond class1="H807" class2="C801" length="0.109000" k="284512.000000"/>
    <Bond class1="H808" class2="C801" length="0.109000" k="284512.000000"/>
  </HarmonicBondForce>
  <HarmonicAngleForce>
    <Angle class1="N800" class2="C801" class3="C802" angle="1.940806" k="669.440000"/>
    <Angle class1="C801" class2="C802" class3="O803" angle="2.042035" k="585.760000"/>
    <Angle class1="C801" class2="C802" class3="O804" angle="2.042035" k="585.760000"/>
    <Angle class1="C801" class2="N800" class3="H805" angle="1.911136" k="292.880000"/>
    <Angle class1="C801" class2="N800" class3="H806" angle="1.911136" k="292.880000"/>
    <Angle class1="N800" class2="C801" class3="H807" angle="1.911136" k="292.880000"/>
    <Angle class1="N800" class2="C801" class3="H808" angle="1.911136" k="292.880000"/>
    <Angle class1="H807" class2="C801" class3="H808" angle="1.881465" k="276.144000"/>
    <Angle class1="H805" class2="N800" class3="H806" angle="1.857030" k="364.844800"/>
    <Angle class1="O803" class2="C802" class3="O804" angle="2.199115" k="669.440000"/>
    <Angle class1="C802" class2="C801" class3="H807" angle="1.911136" k="292.880000"/>
    <Angle class1="C802" class2="C801" class3="H808" angle="1.911136" k="292.880000"/>
  </HarmonicAngleForce>
  <PeriodicTorsionForce>
    <Proper class1="H805" class2="N800" class3="C801" class4="C802" k1="-0.397480" k2="-0.872364"
    k3="0.874456" k4="0.000000" periodicity1="1" periodicity2="2" periodicity3="3"
    periodicity4="4" phase1="0.00" phase2="3.141592653589793" phase3="0.00"
    phase4="3.141592653589793"/>
    <Proper class1="H806" class2="N800" class3="C801" class4="C802" k1="-0.397480" k2="-0.872364"
    k3="0.874456" k4="0.000000" periodicity1="1" periodicity2="2" periodicity3="3"
```

```
    periodicity4="4" phase1="0.00" phase2="3.141592653589793" phase3="0.00"
    phase4="3.141592653589793"/>
    <Proper class1="H807" class2="C801" class3="C802" class4="O803" k1="0.000000" k2="0.000000"
    k3="0.000000" k4="0.000000" periodicity1="1" periodicity2="2" periodicity3="3"
    periodicity4="4" phase1="0.00" phase2="3.141592653589793" phase3="0.00"
    phase4="3.141592653589793"/>
    <Proper class1="H807" class2="C801" class3="C802" class4="O804" k1="0.000000" k2="0.000000"
    k3="0.000000" k4="0.000000" periodicity1="1" periodicity2="2" periodicity3="3"
    periodicity4="4" phase1="0.00" phase2="3.141592653589793" phase3="0.00"
    phase4="3.141592653589793"/>
    <Proper class1="H807" class2="C801" class3="N800" class4="H805" k1="0.000000" k2="0.000000"
    k3="0.836800" k4="0.000000" periodicity1="1" periodicity2="2" periodicity3="3"
    periodicity4="4" phase1="0.00" phase2="3.141592653589793" phase3="0.00"
    phase4="3.141592653589793"/>
    <Proper class1="H807" class2="C801" class3="N800" class4="H806" k1="0.000000" k2="0.000000"
    k3="0.836800" k4="0.000000" periodicity1="1" periodicity2="2" periodicity3="3"
    periodicity4="4" phase1="0.00" phase2="3.141592653589793" phase3="0.00"
    phase4="3.141592653589793"/>
    <Proper class1="H808" class2="C801" class3="C802" class4="O803" k1="0.000000" k2="0.000000"
    k3="0.000000" k4="0.000000" periodicity1="1" periodicity2="2" periodicity3="3"
    periodicity4="4" phase1="0.00" phase2="3.141592653589793" phase3="0.00"
    phase4="3.141592653589793"/>
    <Proper class1="H808" class2="C801" class3="C802" class4="O804" k1="0.000000" k2="0.000000"
    k3="0.000000" k4="0.000000" periodicity1="1" periodicity2="2" periodicity3="3"
    periodicity4="4" phase1="0.00" phase2="3.141592653589793" phase3="0.00"
    phase4="3.141592653589793"/>
    <Proper class1="H808" class2="C801" class3="N800" class4="H805" k1="0.000000" k2="0.000000"
    k3="0.836800" k4="0.000000" periodicity1="1" periodicity2="2" periodicity3="3"
    periodicity4="4" phase1="0.00" phase2="3.141592653589793" phase3="0.00"
    phase4="3.141592653589793"/>
    <Proper class1="H808" class2="C801" class3="N800" class4="H806" k1="0.000000" k2="0.000000"
    k3="0.836800" k4="0.000000" periodicity1="1" periodicity2="2" periodicity3="3"
    periodicity4="4" phase1="0.00" phase2="3.141592653589793" phase3="0.00"
    phase4="3.141592653589793"/>
    <Proper class1="O803" class2="C802" class3="C801" class4="N800" k1="11.003920" k2="1.715440"
    k3="0.000000" k4="0.000000" periodicity1="1" periodicity2="2" periodicity3="3"
    periodicity4="4" phase1="0.00" phase2="3.141592653589793" phase3="0.00"
    phase4="3.141592653589793"/>
    <Proper class1="O804" class2="C802" class3="C801" class4="N800" k1="11.003920" k2="1.715440"
    k3="0.000000" k4="0.000000" periodicity1="1" periodicity2="2" periodicity3="3"
    periodicity4="4" phase1="0.00" phase2="3.141592653589793" phase3="0.00"
    phase4="3.141592653589793"/>
    <Improper class1="N800" class2="C801" class3="H805" class4="H806" k1="0.000000" k2="0.000000"
    k3="0.000000" k4="0.000000" periodicity1="1" periodicity2="2" periodicity3="3"
    periodicity4="4" phase1="0.00" phase2="3.141592653589793" phase3="0.00"
    phase4="3.141592653589793"/>
    <Improper class1="C801" class2="N800" class3="C802" class4="H807" k1="0.000000" k2="0.000000"
    k3="0.000000" k4="0.000000" periodicity1="1" periodicity2="2" periodicity3="3"
    periodicity4="4" phase1="0.00" phase2="3.141592653589793" phase3="0.00"
    phase4="3.141592653589793"/>
    <Improper class1="C801" class2="H808" class3="C802" class4="N800" k1="0.000000" k2="0.000000"
    k3="0.000000" k4="0.000000" periodicity1="1" periodicity2="2" periodicity3="3"
    periodicity4="4" phase1="0.00" phase2="3.141592653589793" phase3="0.00"
    phase4="3.141592653589793"/>
    <Improper class1="C802" class2="C801" class3="O803" class4="O804" k1="0.000000"
    k2="43.932000" k3="0.000000" k4="0.000000" periodicity1="1" periodicity2="2" periodicity3="3"
    periodicity4="4" phase1="0.00" phase2="3.141592653589793" phase3="0.00"
    phase4="3.141592653589793"/>
  </PeriodicTorsionForce>
  <NonbondedForce coulomb14scale="0.5" lj14scale="0.5">
    <Atom type="opls_800" charge="-0.855200" sigma="0.330000" epsilon="0.711280"/>
    <Atom type="opls_805" charge="0.318300" sigma="0.000000" epsilon="0.000000"/>
    <Atom type="opls_806" charge="0.318300" sigma="0.000000" epsilon="0.000000"/>
    <Atom type="opls_807" charge="0.061500" sigma="0.250000" epsilon="0.125520"/>
    <Atom type="opls_802" charge="0.416200" sigma="0.355000" epsilon="0.292880"/>
    <Atom type="opls_808" charge="0.061500" sigma="0.250000" epsilon="0.125520"/>
    <Atom type="opls_804" charge="-0.620900" sigma="0.296000" epsilon="0.878640"/>
    <Atom type="opls_803" charge="-0.620900" sigma="0.296000" epsilon="0.878640"/>
    <Atom type="opls_801" charge="-0.078800" sigma="0.350000" epsilon="0.276144"/>
```

```
  </NonbondedForce>
</ForceField>
```
