# Supplementary material for: Molecular Dynamics Investigation of Clustering in Aqueous Glycine Solutions
Source: J Phys Chem B. 2022 Jun 21;126(25):4711–22. doi: 10.1021/acs.jpcb.2c01975 (PMC9251761; doi:10.1021/acs.jpcb.2c01975)
Supplement: Supplementary file 1 — jp2c01975_si_001.zip [file jp2c01975_si_001.zip › GLC.pdf]

This XML file does not appear to have any style information associated with it. The document tree is shown below.


```xml
<ForceField>
  <AtomTypes>
    <Type name="opls_802" class="H802" element="H" mass="1.008000"/>
    <Type name="opls_809" class="H809" element="H" mass="1.008000"/>
    <Type name="opls_804" class="C804" element="C" mass="12.011000"/>
    <Type name="opls_801" class="H801" element="H" mass="1.008000"/>
    <Type name="opls_805" class="C805" element="C" mass="12.011000"/>
    <Type name="opls_810" class="H810" element="H" mass="1.008000"/>
    <Type name="opls_808" class="H808" element="H" mass="1.008000"/>
    <Type name="opls_803" class="H803" element="H" mass="1.008000"/>
    <Type name="opls_807" class="O807" element="O" mass="15.999000"/>
    <Type name="opls_806" class="O806" element="O" mass="15.999000"/>
    <Type name="opls_800" class="N800" element="N" mass="14.007000"/>
  </AtomTypes>
  <Residues>
    <Residue name="UNK">
      <Atom name="N00" type="opls_800"/>
      <Atom name="H01" type="opls_801"/>
      <Atom name="H02" type="opls_802"/>
      <Atom name="H03" type="opls_803"/>
      <Atom name="C04" type="opls_804"/>
      <Atom name="C05" type="opls_805"/>
      <Atom name="O06" type="opls_806"/>
      <Atom name="O07" type="opls_807"/>
      <Atom name="H08" type="opls_808"/>
      <Atom name="H09" type="opls_809"/>
      <Atom name="H0A" type="opls_810"/>
      <Bond from="0" to="1"/>
      <Bond from="0" to="2"/>
      <Bond from="0" to="3"/>
      <Bond from="0" to="4"/>
      <Bond from="4" to="5"/>
      <Bond from="5" to="6"/>
      <Bond from="5" to="7"/>
      <Bond from="4" to="8"/>
      <Bond from="4" to="9"/>
      <Bond from="7" to="10"/>
    </Residue>
  </Residues>
  <HarmonicBondForce>
    <Bond class1="H801" class2="N800" length="0.101000" k="363171.200000"/>
    <Bond class1="H802" class2="N800" length="0.101000" k="363171.200000"/>
    <Bond class1="H803" class2="N800" length="0.101000" k="363171.200000"/>
    <Bond class1="C804" class2="N800" length="0.147100" k="307105.600000"/>
    <Bond class1="C805" class2="C804" length="0.152200" k="265265.600000"/>
    <Bond class1="O806" class2="C805" length="0.122900" k="476976.000000"/>
    <Bond class1="O807" class2="C805" length="0.136400" k="376560.000000"/>
    <Bond class1="H808" class2="C804" length="0.109000" k="284512.000000"/>
    <Bond class1="H809" class2="C804" length="0.109000" k="284512.000000"/>
    <Bond class1="H810" class2="O807" length="0.094500" k="462750.400000"/>
  </HarmonicBondForce>
  <HarmonicAngleForce>
    <Angle class1="H801" class2="N800" class3="H802" angle="1.911136" k="364.844800"/>
    <Angle class1="H801" class2="N800" class3="H803" angle="1.911136" k="364.844800"/>
    <Angle class1="H801" class2="N800" class3="C804" angle="1.878672" k="269.031200"/>
    <Angle class1="N800" class2="C804" class3="C805" angle="1.940806" k="669.440000"/>
    <Angle class1="C804" class2="C805" class3="O806" angle="2.101376" k="669.440000"/>
    <Angle class1="C804" class2="C805" class3="O807" angle="1.884956" k="585.760000"/>
    <Angle class1="N800" class2="C804" class3="H808" angle="1.911136" k="292.880000"/>
    <Angle class1="N800" class2="C804" class3="H809" angle="1.911136" k="292.880000"/>
    <Angle class1="C805" class2="O807" class3="H810" angle="1.972222" k="292.880000"/>
    <Angle class1="C805" class2="C804" class3="H808" angle="1.911136" k="292.880000"/>
    <Angle class1="H802" class2="N800" class3="H803" angle="1.911136" k="364.844800"/>
    <Angle class1="H808" class2="C804" class3="H809" angle="1.881465" k="276.144000"/>
    <Angle class1="H803" class2="N800" class3="C804" angle="1.878672" k="269.031200"/>
```

```xml
    <Angle class1="H802" class2="N800" class3="C804" angle="1.878672" k="269.031200"/>
    <Angle class1="C805" class2="C804" class3="H809" angle="1.911136" k="292.880000"/>
    <Angle class1="O806" class2="C805" class3="O807" angle="2.111848" k="669.440000"/>
  </HarmonicAngleForce>
  <PeriodicTorsionForce>
    <Proper class1="C805" class2="C804" class3="N800" class4="H801" k1="0.000000" k2="0.000000"
    k3="0.725924" k4="0.000000" periodicity1="1" periodicity2="2" periodicity3="3"
    periodicity4="4" phase1="0.00" phase2="3.141592653589793" phase3="0.00"
    phase4="3.141592653589793"/>
    <Proper class1="C805" class2="C804" class3="N800" class4="H802" k1="0.000000" k2="0.000000"
    k3="0.725924" k4="0.000000" periodicity1="1" periodicity2="2" periodicity3="3"
    periodicity4="4" phase1="0.00" phase2="3.141592653589793" phase3="0.00"
    phase4="3.141592653589793"/>
    <Proper class1="C805" class2="C804" class3="N800" class4="H803" k1="0.000000" k2="0.000000"
    k3="0.725924" k4="0.000000" periodicity1="1" periodicity2="2" periodicity3="3"
    periodicity4="4" phase1="0.00" phase2="3.141592653589793" phase3="0.00"
    phase4="3.141592653589793"/>
    <Proper class1="H808" class2="C804" class3="C805" class4="O806" k1="0.000000" k2="0.000000"
    k3="0.000000" k4="0.000000" periodicity1="1" periodicity2="2" periodicity3="3"
    periodicity4="4" phase1="0.00" phase2="3.141592653589793" phase3="0.00"
    phase4="3.141592653589793"/>
    <Proper class1="H808" class2="C804" class3="C805" class4="O807" k1="0.000000" k2="0.000000"
    k3="0.000000" k4="0.000000" periodicity1="1" periodicity2="2" periodicity3="3"
    periodicity4="4" phase1="0.00" phase2="3.141592653589793" phase3="0.00"
    phase4="3.141592653589793"/>
    <Proper class1="H808" class2="C804" class3="N800" class4="H801" k1="0.000000" k2="0.000000"
    k3="0.546012" k4="0.000000" periodicity1="1" periodicity2="2" periodicity3="3"
    periodicity4="4" phase1="0.00" phase2="3.141592653589793" phase3="0.00"
    phase4="3.141592653589793"/>
    <Proper class1="H808" class2="C804" class3="N800" class4="H802" k1="0.000000" k2="0.000000"
    k3="0.546012" k4="0.000000" periodicity1="1" periodicity2="2" periodicity3="3"
    periodicity4="4" phase1="0.00" phase2="3.141592653589793" phase3="0.00"
    phase4="3.141592653589793"/>
    <Proper class1="H808" class2="C804" class3="N800" class4="H803" k1="0.000000" k2="0.000000"
    k3="0.546012" k4="0.000000" periodicity1="1" periodicity2="2" periodicity3="3"
    periodicity4="4" phase1="0.00" phase2="3.141592653589793" phase3="0.00"
    phase4="3.141592653589793"/>
    <Proper class1="H809" class2="C804" class3="C805" class4="O806" k1="0.000000" k2="0.000000"
    k3="0.000000" k4="0.000000" periodicity1="1" periodicity2="2" periodicity3="3"
    periodicity4="4" phase1="0.00" phase2="3.141592653589793" phase3="0.00"
    phase4="3.141592653589793"/>
    <Proper class1="H809" class2="C804" class3="C805" class4="O807" k1="0.000000" k2="0.000000"
    k3="0.000000" k4="0.000000" periodicity1="1" periodicity2="2" periodicity3="3"
    periodicity4="4" phase1="0.00" phase2="3.141592653589793" phase3="0.00"
    phase4="3.141592653589793"/>
    <Proper class1="H809" class2="C804" class3="N800" class4="H801" k1="0.000000" k2="0.000000"
    k3="0.546012" k4="0.000000" periodicity1="1" periodicity2="2" periodicity3="3"
    periodicity4="4" phase1="0.00" phase2="3.141592653589793" phase3="0.00"
    phase4="3.141592653589793"/>
    <Proper class1="H809" class2="C804" class3="N800" class4="H802" k1="0.000000" k2="0.000000"
    k3="0.546012" k4="0.000000" periodicity1="1" periodicity2="2" periodicity3="3"
    periodicity4="4" phase1="0.00" phase2="3.141592653589793" phase3="0.00"
    phase4="3.141592653589793"/>
    <Proper class1="H809" class2="C804" class3="N800" class4="H803" k1="0.000000" k2="0.000000"
    k3="0.546012" k4="0.000000" periodicity1="1" periodicity2="2" periodicity3="3"
    periodicity4="4" phase1="0.00" phase2="3.141592653589793" phase3="0.00"
    phase4="3.141592653589793"/>
    <Proper class1="H810" class2="O807" class3="C805" class4="C804" k1="3.138000" k2="11.506000"
    k3="0.000000" k4="0.000000" periodicity1="1" periodicity2="2" periodicity3="3"
    periodicity4="4" phase1="0.00" phase2="3.141592653589793" phase3="0.00"
    phase4="3.141592653589793"/>
    <Proper class1="H810" class2="O807" class3="C805" class4="O806" k1="0.000000" k2="11.506000"
    k3="0.000000" k4="0.000000" periodicity1="1" periodicity2="2" periodicity3="3"
    periodicity4="4" phase1="0.00" phase2="3.141592653589793" phase3="0.00"
    phase4="3.141592653589793"/>
    <Proper class1="O806" class2="C805" class3="C804" class4="N800" k1="0.000000" k2="0.000000"
    k3="0.000000" k4="0.000000" periodicity1="1" periodicity2="2" periodicity3="3"
    periodicity4="4" phase1="0.00" phase2="3.141592653589793" phase3="0.00"
    phase4="3.141592653589793"/>
```

```
    <Proper class1="O807" class2="C805" class3="C804" class4="N800" k1="11.003920" k2="1.715440"
    k3="0.000000" k4="0.000000" periodicity1="1" periodicity2="2" periodicity3="3"
    periodicity4="4" phase1="0.00" phase2="3.141592653589793" phase3="0.00"
    phase4="3.141592653589793"/>
    <Improper class1="N800" class2="H801" class3="H802" class4="C804" k1="0.000000" k2="0.000000"
    k3="0.000000" k4="0.000000" periodicity1="1" periodicity2="2" periodicity3="3"
    periodicity4="4" phase1="0.00" phase2="3.141592653589793" phase3="0.00"
    phase4="3.141592653589793"/>
    <Improper class1="N800" class2="H801" class3="H802" class4="H803" k1="0.000000" k2="0.000000"
    k3="0.000000" k4="0.000000" periodicity1="1" periodicity2="2" periodicity3="3"
    periodicity4="4" phase1="0.00" phase2="3.141592653589793" phase3="0.00"
    phase4="3.141592653589793"/>
    <Improper class1="C804" class2="H808" class3="N800" class4="C805" k1="0.000000" k2="0.000000"
    k3="0.000000" k4="0.000000" periodicity1="1" periodicity2="2" periodicity3="3"
    periodicity4="4" phase1="0.00" phase2="3.141592653589793" phase3="0.00"
    phase4="3.141592653589793"/>
    <Improper class1="C804" class2="N800" class3="H809" class4="C805" k1="0.000000" k2="0.000000"
    k3="0.000000" k4="0.000000" periodicity1="1" periodicity2="2" periodicity3="3"
    periodicity4="4" phase1="0.00" phase2="3.141592653589793" phase3="0.00"
    phase4="3.141592653589793"/>
    <Improper class1="C805" class2="C804" class3="O806" class4="O807" k1="0.000000"
    k2="43.932000" k3="0.000000" k4="0.000000" periodicity1="1" periodicity2="2" periodicity3="3"
    periodicity4="4" phase1="0.00" phase2="3.141592653589793" phase3="0.00"
    phase4="3.141592653589793"/>
  </PeriodicTorsionForce>
  <NonbondedForce coulomb14scale="0.5" lj14scale="0.5">
    <Atom type="opls_802" charge="0.366400" sigma="0.000000" epsilon="0.000000"/>
    <Atom type="opls_804" charge="-0.171400" sigma="0.350000" epsilon="0.276144"/>
    <Atom type="opls_801" charge="0.366400" sigma="0.000000" epsilon="0.000000"/>
    <Atom type="opls_810" charge="0.432400" sigma="0.000000" epsilon="0.000000"/>
    <Atom type="opls_807" charge="-0.420500" sigma="0.312000" epsilon="0.711280"/>
    <Atom type="opls_808" charge="0.170600" sigma="0.250000" epsilon="0.125520"/>
    <Atom type="opls_805" charge="0.416100" sigma="0.355000" epsilon="0.292880"/>
    <Atom type="opls_809" charge="0.170600" sigma="0.250000" epsilon="0.125520"/>
    <Atom type="opls_803" charge="0.366400" sigma="0.000000" epsilon="0.000000"/>
    <Atom type="opls_800" charge="-0.373500" sigma="0.325000" epsilon="0.711280"/>
    <Atom type="opls_806" charge="-0.323400" sigma="0.296000" epsilon="0.878640"/>
  </NonbondedForce>
</ForceField>
```
